# The Role of Dicer Phosphorylation in Gemcitabine Resistance of Pancreatic Cancer

**DOI:** 10.3390/ijms252111797

**Published:** 2024-11-02

**Authors:** Ching-Feng Chiu, Hui-Ru Lin, Yen-Hao Su, Hsin-An Chen, Shao-Wen Hung, Shih-Yi Huang

**Affiliations:** 1 Graduate Institute of Metabolism and Obesity Sciences, Taipei Medical University, Taipei 110, Taiwan; chiucf@tmu.edu.tw (C.-F.C.); ma48111003@tmu.edu.tw (H.-R.L.); 2 Division of General Surgery, Shuang Ho Hospital, Taipei Medical University, Taipei 110, Taiwan; su11066@tmu.edu.tw (Y.-H.S.); d118101004@tmu.edu.tw (H.-A.C.); 3 Division of Animal Industry, Animal Technology Laboratories, Agricultural Technology Research Institute, Xiangshan Dist., Hsinchu City 300, Taiwan; 1032169@mail.atri.org.tw; 4 School of Nutrition and Health Sciences, Taipei Medical University, Taipei 110, Taiwan

**Keywords:** phosphorylated Dicer, microRNA, glutamine, pancreatic ductal adenocarcinoma, gemcitabine

## Abstract

Dicer, a cytoplasmic type III RNase, is essential for the maturation of microRNAs (miRNAs) and is implicated in cancer progression and chemoresistance. Our previous research demonstrated that phosphorylation of Dicer at S1016 alters miRNA maturation and glutamine metabolism, contributing to gemcitabine (GEM) resistance in pancreatic ductal adenocarcinoma (PDAC). In this study, we focused on the role of Dicer phosphorylation at S1728/S1852 in GEM-resistant PDAC cells. Using shRNA to knock down Dicer in GEM-resistant PANC-1 (PANC-1 GR) cells, we examined cell viability through MTT and clonogenic assays. We also expressed phosphomimetic Dicer 2E (S1728E/S1852E) and phosphomutant Dicer 2A (S1728A/S1852A) to evaluate their effects on GEM resistance and metabolism. Our results show that phosphorylation at S1728/S1852 promotes GEM resistance by reprogramming glutamine metabolism. Specifically, phosphomimetic Dicer 2E increased intracellular glutamine, driving pyrimidine synthesis and raising dCTP levels, which compete with gemcitabine’s metabolites. This metabolic shift enhanced drug resistance. In contrast, phosphomutant Dicer 2A reduced GEM resistance. These findings highlight the importance of Dicer phosphorylation in regulating metabolism and drug sensitivity, offering insights into potential therapeutic strategies for overcoming GEM resistance in pancreatic cancer.

## 1. Introduction

Pancreatic cancer is a common malignant tumor with a poor prognosis [1]. Furthermore, pancreatic cancer is usually diagnosed at a late stage, resulting in a meager five-year survival rate of only 9% [2]. This is attributed to the early tendency of pancreatic cancer to metastasize and its aggressive local growth, leading to fewer than 20% of patients being eligible for surgical resection. Consequently, chemotherapy is a crucial treatment method for pancreatic cancer [3]. Common drugs include gemcitabine (GEM), used alone or combined with modified FOLFIRINOX (modified folic acid, Fluorouracil, Irinotecan, and Oxaliplatin). However, clinical cases show that many pancreatic cancer patients have developed resistance to GEM. Therefore, the exact mechanisms need further research to identify the causes of drug resistance and improve treatment efficacy.

Dicer is a type III ribonuclease (RNase) in the biosynthesis pathway of miRNA and a crucial regulator of post-transcriptional gene silencing [4]. Previous studies have indicated that abnormal expression of Dicer is associated with cancer progression and metastasis [5,6,7]. Our laboratory’s prior research has also found that ERK-mediated SP1 transcription factor binds to the Dicer promoter region and induces increased Dicer expression, contributing to the survival of gemcitabine-resistant pancreatic cancer cells [8]. The mechanism underlying Dicer-induced cancer growth and drug resistance is related to the phosphorylation of Dicer. Studies have indicated that phosphorylation of Dicer at S1728 and S1852 promotes tumor growth and metastasis or even triggers the DNA damage response (DDR) as a protective mechanism [9,10]. Therefore, we found that phosphomimetic Dicer S1016E increased the GLS/GLUL ratio by affecting specific miRNAs, altering glutamine metabolism, and enhancing gemcitabine resistance [11]. However, further research is needed to confirm the pathways related to the phosphorylation of Dicer at the S1728 and S1852 sites and their impact on drug resistance.

Metabolic abnormalities are a significant hallmark of cancer, including not only increased aerobic glycolysis but also glutamine anaplerosis, also called glutamine addition. These metabolic abnormalities impact tumorigenesis, metastasis, chemoresistance, and cancer stem cells [12,13]. Therefore, disruption of the glutamine metabolic pathway has been shown to enhance the efficacy of gemcitabine treatment in gemcitabine-resistant pancreatic cancer cells [14]. Additionally, miR-140-5p regulation of GLUL (glutamine synthetase) expression in glioma cells inhibits cancer cell proliferation, migration, and invasion [15]. This suggests that miRNAs may be involved in metabolic shifts in cancer cells.

This study explored the mechanism of Dicer phosphorylation involved in GEM resistance and metabolic reprogramming in PDAC. We previously found that phosphomimetic Dicer S1016E increased the GLS/GLUL ratio by affecting specific miRNAs, altering glutamine metabolism, and enhancing gemcitabine resistance [11]. In this study, we further investigated the effect of Dicer phosphorylation at S1728/1852 and its association with glutamine metabolism and chemotherapy responses in GEM-resistant PDAC cells. The results revealed that re-overexpression of phosphomimetic Dicer at S1728E/S1852E in Dicer-silenced GR cells altered glutamine metabolism by reducing the expression of specific miRNAs, leading to the accumulation of intracellular glutamine as a substrate for pyrimidine synthesis. This increases dCTP and molecular competition with the final metabolites produced by gemcitabine, contributing to gemcitabine resistance.

## 2. Results

### 2.1. Dicer Expression in PDAC Tumor Tissue and Its Impact on Survival Rate

**Figure 1:**
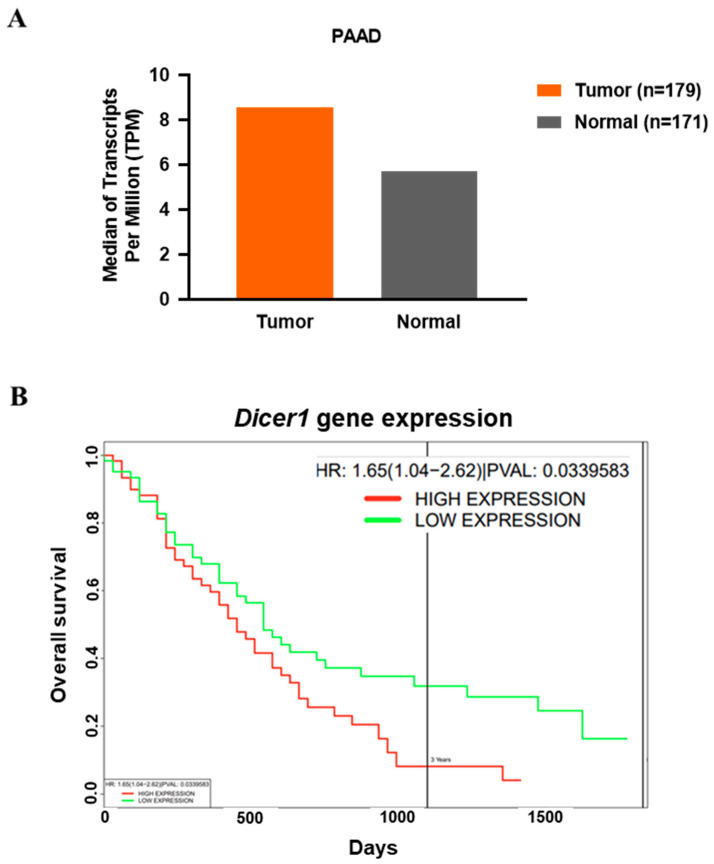
The expression level of *Dicer1* in PDAC tumor tissues and its impact on survival rates. (**A**) Analysis of the difference in *Dicer1* gene expression between PDAC tumor tissues (PAAD, n = 179) and normal tissues (n = 171) using the online database GEPIA; (**B**) survival analysis of pancreatic cancer patients based on *Dicer* expression levels (red line: high expression; green line: low expression) using the PROGgeneV2 Prognostic Database.

The online database GEPIA (Gene Expression Profiling Interactive Analysis) found that *Dicer* expression levels are higher in pancreatic cancer tumor tissues than in normal tissues (Figure 1A). Additionally, analysis of another online database, PROGgeneV2 Prognostic Database, revealed that pancreatic cancer patients with low *Dicer* expression have a significantly higher overall survival rate after three years compared to patients with high Dicer expression (*p* < 0.05) (Figure 1B).

### 2.2. High Dicer Expression Affects Pancreatic Cancer Cell Proliferation and the Development of Gemcitabine Resistance

**Figure 2:**
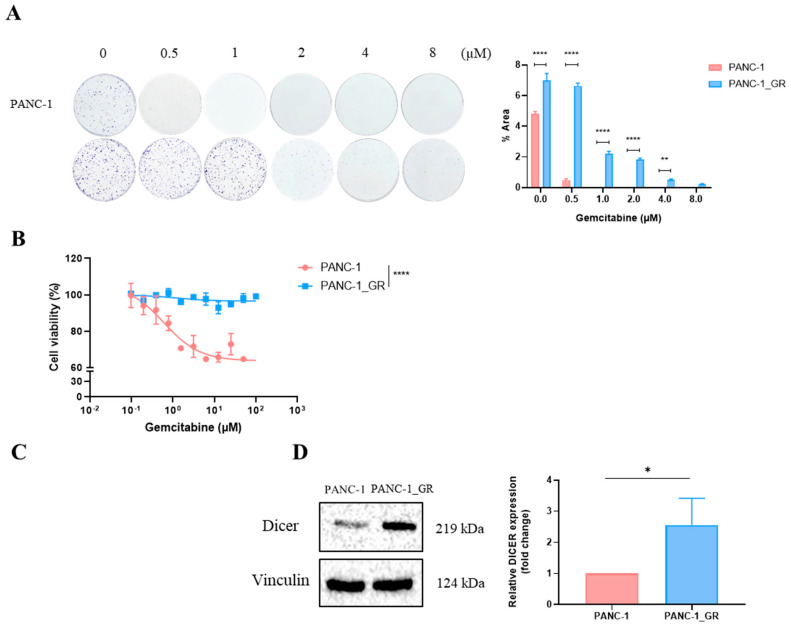
The expression level of Dicer in the pancreatic cancer cell line PANC-1 GR is positively correlated with gemcitabine resistance and cell proliferation. (**A**) The clonogenicity assay was used to test colony formation ability under different concentrations of gemcitabine treatment for 7 days. (**B**) The MTT assay was used to test cell viability after treatment with different concentrations of gemcitabine for 72 h. Data are presented as mean ± SEM and analyzed using Student’s *t*-test. (**C**) The mRNA expression level of Dicer in PANC-1 and PANC-1 GR cells was tested using qRT-PCR. The qRT-PCR data were normalized to the GAPGH level in each sample, and a bar plot presents fold changes in the expression of PANC-1 cells. (**D**) The protein expression level of Dicer in PANC-1 and PANC-1 GR cells was tested using Western Blot, with Vinculin as the control. Data are presented as mean ± SEM and analyzed using Student’s *t*-test. Significant differences are indicated when *p* < 0.05 (represented as * *p* < 0.05, ** *p* < 0.01, and **** *p* < 0.0001).

Cell growth was observed after one week of gemcitabine treatment through a colony formation assay. The results showed that the colony area of PANC-1 GR cells was significantly more extensive than that of PANC-1 cells (*p* < 0.0001) (Figure 2A). Furthermore, cell viability analysis showed that after 72 h of gemcitabine treatment, the survival rate of PANC-1 GR cells was significantly higher than that of PANC-1 cells (*p* < 0.0001) (Figure 2B). These results indicate that this experiment’s PANC-1 GR cell line exhibits gemcitabine resistance. Subsequent qRT-PCR and Western Blot analyses revealed that the expression level of Dicer in the gemcitabine-resistant PANC-1 GR cell line was significantly higher than that in PANC-1 cells (*p* < 0.05) (Figure 2C,D). Therefore, these results suggest a positive correlation between Dicer expression levels and gemcitabine resistance.

**Figure 3:**
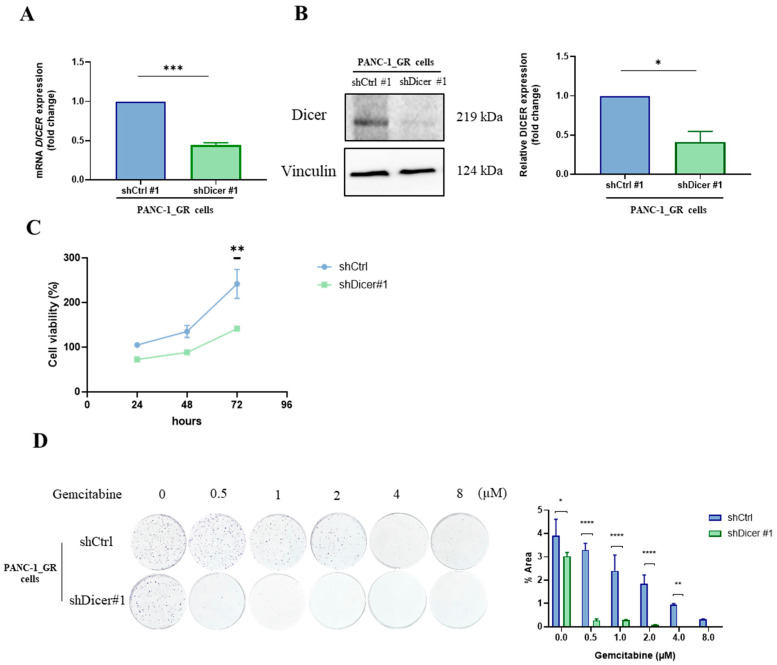
Silencing Dicer expression in the pancreatic cancer cell line PANC-1 GR increases sensitivity to gemcitabine and reduces cell growth rate. (**A**) The mRNA expression level of Dicer in PANC-1 GR/shCtrl #1 and PANC-1 GR/shDicer#1 cells was tested using qRT-PCR. The qRT-PCR data were normalized to the GAPGH level in each sample, and a bar plot presents fold changes in the expression of PANC-1_GR_shCtrl#1 cells. (**B**) The protein expression level of Dicer in PANC-1 GR/shCtrl #1 and PANC-1 GR/shDicer#1 cells was tested using Western Blot, with Vinculin as the control. (**C**) The MTT assay was used to test cell viability at 24, 48, and 72 h in PANC-1 GR/shCtrl #1 and PANC-1 GR/shDicer#1 cells. (**D**) The clonogenicity assay was used to test colony formation ability under different concentrations of gemcitabine treatment for 7 days. Data are presented as mean ± SEM and analyzed using Student’s *t*-test and Two-way ANOVA. Significant differences are indicated when *p* < 0.05 (represented as * *p* < 0.05, ** *p* < 0.01, *** *p* < 0.001, and **** *p* < 0.0001).

To further investigate whether Dicer expression affects the development of gemcitabine resistance in pancreatic cancer cells, we established PANC-1 GR cells with silenced Dicer using lentiviral infection. We confirmed the silencing effect through qRT-PCR and Western Blot (Figure 3A,B). Additionally, cell viability assays comparing the growth characteristics of the two cell lines showed that at 72 h, the growth rate of PANC-1 GR/shDicer was significantly lower than that of PANC-1 GR/shCtrl (*p* < 0.01), with a similar trend at 24 and 48 h (Figure 3C). Moreover, as the dose of gemcitabine increased, the colony area of PANC-1 GR/shDicer cells was significantly smaller than that of PANC-1 GR/shCtrl, indicating a significant increase in drug sensitivity to gemcitabine in PANC-1 GR/shDicer cells (*p* < 0.0001) (Figure 3D). This demonstrates that high expression of Dicer enhances gemcitabine resistance in pancreatic cancer cells and increases the cell proliferation rate.

### 2.3. The Association Between Phosphorylated Dicer and Gemcitabine-Resistant Pancreatic Cancer Cells

**Figure 4:**
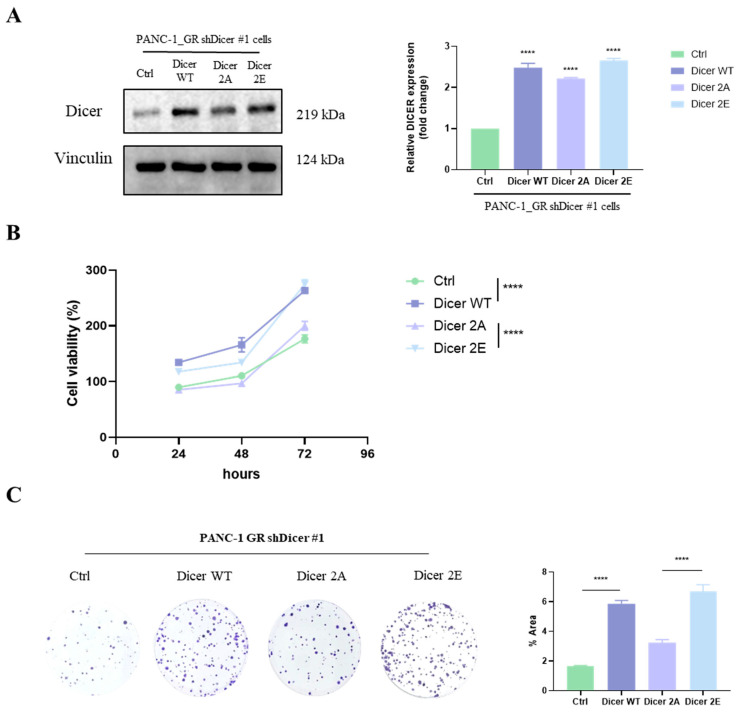
Simulating phosphorylation of Dicer at S1728E/S1852E in PANC-1 GR/shDicer cells restores cell growth rate. (**A**) The protein expression level of Dicer wild type (WT), phosphomutant Dicer S1728A/S1852A, and phosphomimetic Dicer S1728E/S1852E was tested using Western Blot, with Vinculin as the control. (**B**) The MTT assay was used to test cell viability at 24, 48, and 72 h in Ctrl, Dicer WT, Dicer 2A, and Dicer 2E cells. (**C**) The clonogenicity assay was used to test colony formation ability under different concentrations of gemcitabine treatment for 7 days in Ctrl, Dicer WT, Dicer 2A, and Dicer 2E cells. Data are presented as mean ± SEM and analyzed using One-way ANOVA and Two-way ANOVA. Significant differences are indicated when *p* < 0.05 (represented as **** *p* < 0.0001).

To investigate the role of Dicer phosphorylation at S1728 and S1852 sites in gemcitabine-resistant pancreatic cancer cells, we transfected PANC-1 GR/shDicer cells to express phosphomimetic Dicer S1728E/S1852E and phosphomutant Dicer S1728A/S1852A. We examined the effects of phosphorylated Dicer on colony formation and gemcitabine sensitivity. First, Western Blot was used to confirm successful transfection, and the results showed that the expression levels of overexpressed Dicer wild type (WT), Dicer S1728A/S1852A (2A), and Dicer S1728E/S1852E (2E) were significantly higher than those of the control group (*p* < 0.05). Additionally, the expression levels of Dicer 2E and Dicer 2A were not substantially different from Dicer WT (*p* > 0.05) (Figure 4A).

Next, we observed the cell growth rate after gemcitabine treatment. At 24, 48, and 72 h, the growth rates of Dicer WT and Dicer 2E were significantly higher than those of the control and Dicer 2A (*p* < 0.0001) (Figure 4B). Furthermore, the colony formation assay revealed that Dicer WT and Dicer 2E significantly restored the ability to form colonies compared to the control group. At the same time, Dicer 2A continued to inhibit cell growth (*p* < 0.0001) (Figure 4C). These results demonstrate that phosphorylation at Dicer S1728E/S1852E can increase the rate of cell proliferation.

**Figure 5:**
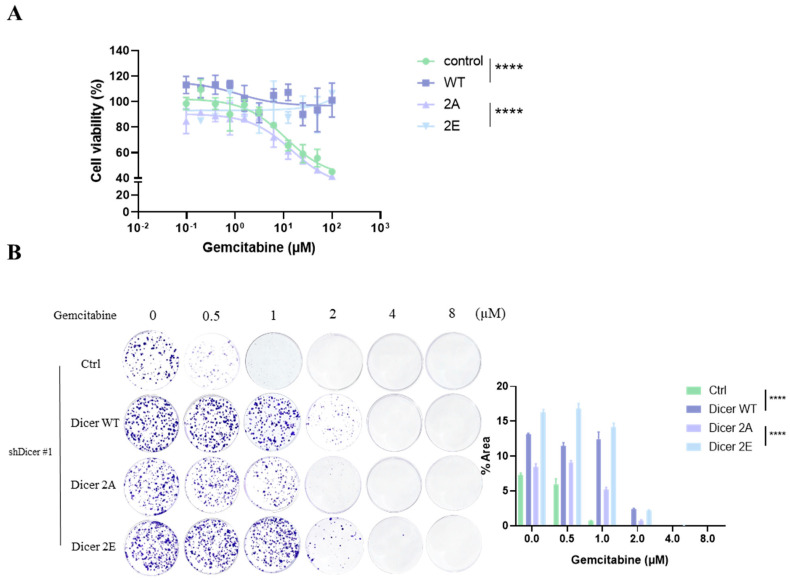
Simulating phosphorylation of Dicer at S1728E/S1852E in PANC-1 GR/shDicer cells restores resistance to gemcitabine. (**A**) The MTT assay was used to test cell viability after treatment with different concentrations of gemcitabine in Ctrl, Dicer WT, Dicer 2A, and Dicer 2E cells after 72 h. (**B**) The clonogenicity assay was used to test colony formation ability under different concentrations of gemcitabine treatment in Ctrl, Dicer WT, Dicer 2A, and Dicer 2E cells after 7 days. Data are presented as mean ± SEM and analyzed using One-way ANOVA. Significant differences are indicated when *p* < 0.05 (represented as and **** *p* < 0.0001).

Subsequently, we treated the cells with different concentrations of gemcitabine to observe drug sensitivity over three days and one week. The results showed that, compared to the control and Dicer 2A, the cell viability of Dicer WT and Dicer 2E remained significantly higher under both high-dose gemcitabine treatment for three days (10 µM, Figure 5A) and lower-dose therapy for one week (Figure 5B), indicating retained drug resistance. In contrast, Dicer 2A lost its resistance to gemcitabine. These findings suggest that phosphorylation at Dicer S1728E/S1852E is associated with developing gemcitabine resistance and changes in cell growth characteristics in pancreatic cancer cells.

### 2.4. Phosphorylated Dicer Influences Glutamine Metabolism Balance in Pancreatic Cancer Cells by Regulating Specific miRNAs, Thereby Increasing Intracellular Glutamine Levels

**Figure 6:**
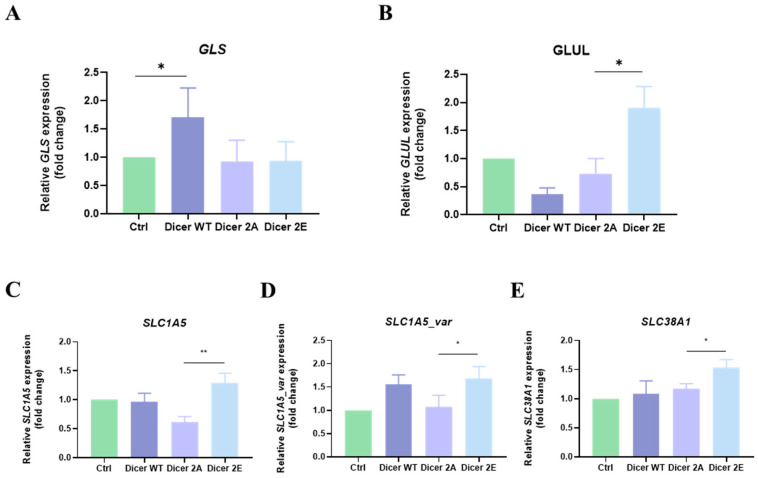
Impact of simulating phosphorylation of Dicer S1728E/S1852E on glutamine metabolism in PANC-1 GR/shDicer cells. (**A**–**E**) The mRNA expression levels of GLS, GLUL, SLC38A1, SLC1A5, and SLC1A5_var in Ctrl, Dicer WT, Dicer 2A, and Dicer 2E cells were tested using qRT-PCR. The qRT-PCR data were normalized to the GAPGH level in each sample, and a bar plot presents fold changes in the expression of PANC-1_GR_shCtrl#1 cells. Data are presented as mean ± SEM and analyzed using One-way ANOVA. Significant differences are indicated when *p* < 0.05 (represented as * *p* < 0.05, ** *p* < 0.01).

Previous studies have shown that the development of gemcitabine resistance in pancreatic cancer cells is closely related to abnormal cancer cell metabolism. Furthermore, based on our past research, phosphomimetic Dicer phosphorylation at another site (S1016E) affected glutamine metabolism in pancreatic cancer cells, leading to gemcitabine resistance. Therefore, we examined the change in metabolism-related genes. The results of qPCR showed that the expression of GLUL was significantly increased in Dicer 2E (*p* < 0.05), while there were no changes in GLS and GLUL expression in the control group and Dicer 2A (Figure 6A,B). Additionally, observing changes in the expression of transport proteins involved in glutamine metabolism, compared to Dicer 2A, Dicer 2E had significantly higher expressions of SLC1A5 (*p* < 0.01) (Figure 6C), SLC38A1 (*p* < 0.05) (Figure 6D), and SLC1A5_var (*p* < 0.05) (Figure 6E).

**Figure 7:**
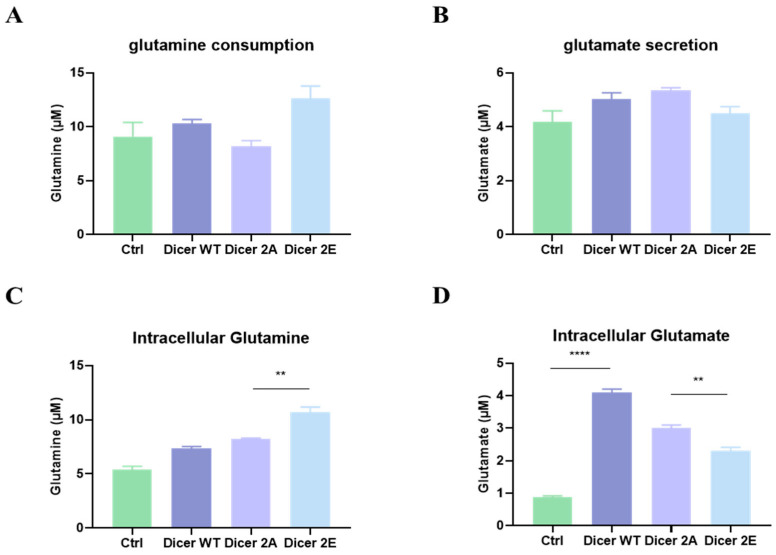
Simulating Dicer S1728E/S1852E phosphorylation in PANC-1 GR/shDicer cells impacts glutamine metabolism, further increasing intracellular glutamine concentration. (**A**–**D**) The intracellular concentrations of glutamine and glutamate and the concentrations in the culture medium were measured using the glutamine/glutamate-Glo assay after 72 h. Data are presented as mean ± SEM and analyzed using One-way ANOVA. Significant differences are indicated when *p* < 0.05 (represented as ** *p* < 0.01, **** *p* < 0.0001).

Moreover, using the glutamine/glutamate-Glo assay kit to detect the cells’ ability to uptake glutamine from the medium and release glutamate, the results showed that Dicer 2E had a higher capacity to uptake glutamine and a trend of releasing less glutamate into the extracellular environment (Figure 7A,B). On the other hand, the intracellular glutamine concentration in Dicer 2E was significantly higher than in Dicer 2A (*p* < 0.01) (Figure 7C). In comparison, the intracellular glutamate concentration was considerably lower than in Dicer 2A (*p* < 0.01) (Figure 7D). These results correspond with the significant increases in GLUL expression and the expression of glutamine metabolism transport proteins in Dicer 2E, indicating that phosphorylation at Dicer S1728E/S1852E significantly increases GLUL expression and the expression of glutamine metabolism transport proteins, further increasing the intracellular glutamine concentration and accumulating within the cells.

**Figure 8:**
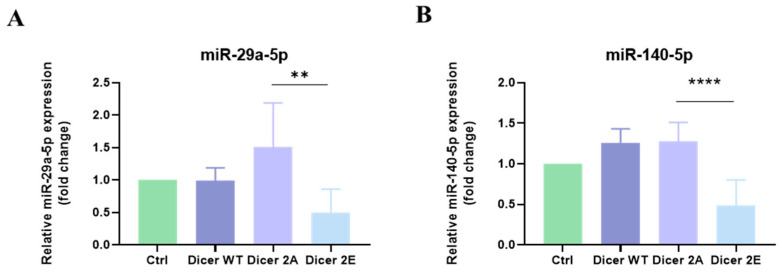
Simulating Dicer S1728E/S1852E phosphorylation in PANC-1 GR/shDicer cells regulates specific miRNAs affecting glutamine metabolism. (**A**,**B**) The expression levels of miRNAs regulating GLUL in Ctrl, Dicer WT, Dicer 2A, and Dicer 2E cells were tested using qRT-PCR. The qRT-PCR data were normalized to the U47 level in each sample, and a bar plot presents fold changes in the expression of PANC-1_GR_shCtrl#1 cells. Data are presented as mean ± SEM and analyzed using One-way ANOVA. Significant differences are indicated when *p* < 0.05 (represented as ** *p* < 0.01, **** *p* < 0.0001).

Referring to previous studies and using TargetScan (http://www.targetscan.org/vert_72/, accessed on 20 August 2024) software analysis, miR-29a-5p and miR-140-5p can bind to glutamine synthetase (GLUL) and regulate mRNA and protein expression [11]. To understand how phosphorylation at Dicer S1728E/S1852E regulates GLUL, we designed primers for miR-29a-5p and miR-140-5p and used qPCR to detect changes in these miRNAs. The results showed that the expression levels of miR-29a-5p and miR-140-5p in Dicer 2E were significantly lower than in Dicer 2A (*p* < 0.01) (Figure 8A,B). This confirms that the expression of GLUL is increased substantially in Dicer 2E (Figure 8A,B), demonstrating that phosphorylation at Dicer S1728E/S1852E affects the expression of glutamine synthetase in pancreatic cancer cells through the regulation of miR-29a-5p and miR-140-5p, thereby influencing glutamine metabolism balance and further increasing intracellular glutamine concentration.

### 2.5. Addressing the Abnormal Glutamine Metabolism in Gemcitabine-Resistant Pancreatic Cancer Cells Further Inhibits Cell Proliferation and Increases Drug Sensitivity to Gemcitabine

**Figure 9:**
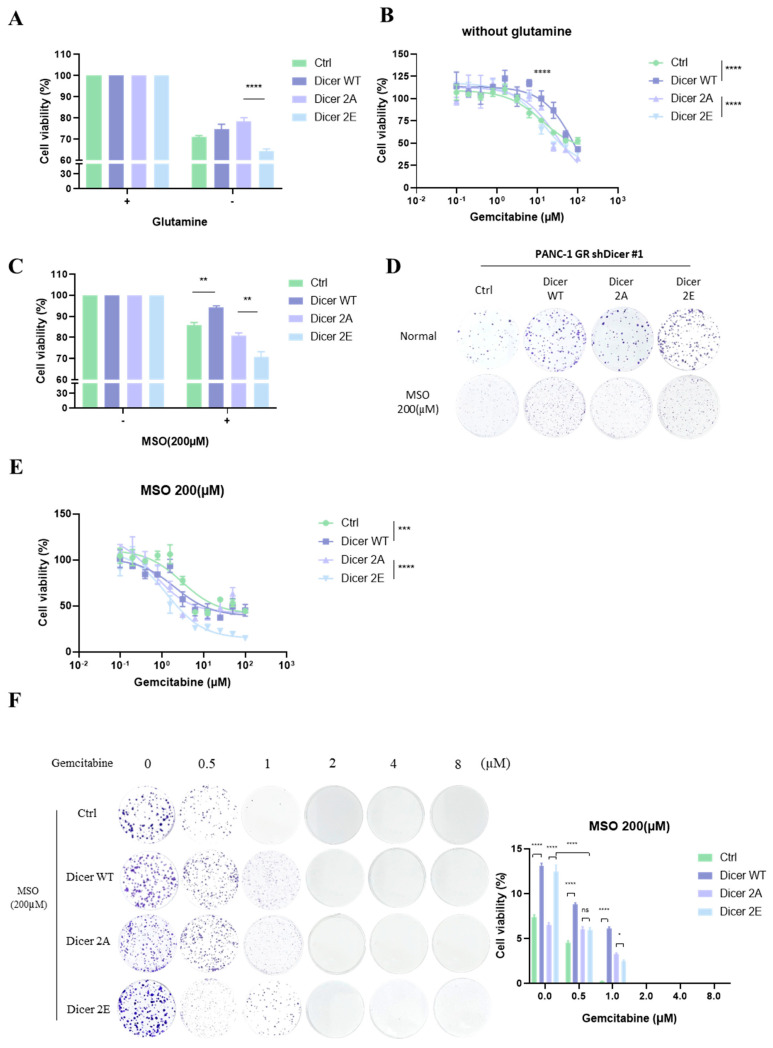
Inhibition of cell proliferation and increased sensitivity to gemcitabine through resolving glutamine metabolism abnormalities in pancreatic cancer drug-resistant cells. (**A**) The MTT assay was used to test cell viability after Ctrl, Dicer WT, Dicer 2A, and Dicer 2E cells were cultured in a glutamine-free medium after 72 h. (**B**) The MTT assay was used to test cell viability, followed by treatment with different concentrations of gemcitabine after 72 h, of Ctrl, Dicer WT, Dicer 2A, and Dicer 2E cells cultured in glutamine-free medium. (**C**) The MTT assay was used to test the cell viability of Ctrl, Dicer WT, Dicer 2A, and Dicer 2E cells treated with methionine sulfoximine (MSO) after 72 h. (**D**) The clonogenicity assay was used to test Ctrl, Dicer WT, Dicer 2A, and Dicer 2E cells’ colony formation ability after being treated with methionine sulfoximine (MSO) after 7 days. (**E**) Three thousand cells/well were seeded in a 96-well plate and treated with methionine sulfoximine (MSO), followed by treatment with different concentrations of gemcitabine, and MTT assay to test the cell viability of Ctrl, Dicer WT, Dicer 2A, and Dicer 2E cells after 72 h. (**F**) Five hundred cells/well were seeded in a 6-well plate and treated with methionine sulfoximine (MSO), followed by clonogenicity assay to test the colony formation ability of Ctrl, Dicer WT, Dicer 2A, and Dicer 2E cells under different concentrations of gemcitabine treatment after 7 days. Data are presented as mean ± SEM and analyzed using One-way and Two-way ANOVAs. Significant differences are indicated when *p* < 0.05 (represented as * *p* < 0.05, ** *p* < 0.01, *** *p* < 0.001, **** *p* < 0.0001, and ns: Non significance.).

To confirm that phosphorylation of Dicer at S1728E/S1852E leads to the development of gemcitabine resistance and affects cancer cell proliferation by altering glutamine metabolism balance, we cultured the cells in a glutamine-deficient medium. We used cell viability assays and colony formation assays to determine whether glutamine deficiency can slow down the growth of pancreatic cancer cells and increase their sensitivity to gemcitabine. The results showed that consistent with the qPCR results, Dicer 2E, which had the highest intracellular glutamine concentration, exhibited significantly reduced cell proliferation under glutamine-deficient conditions (*p* < 0.0001) and significantly increased sensitivity to gemcitabine (*p* < 0.0001) compared to Dicer 2A (Figure 9A,B).

Additionally, we treated the cells with the GLUL inhibitor methionine sulfoximine (MSO). We used cell viability and colony formation assays to determine whether inhibiting GLUL activity affects pancreatic cancer cell growth and gemcitabine sensitivity. Similarly consistent with the qPCR results, Dicer 2E, which had higher GLUL expression, showed significantly reduced cell proliferation over three days or one week after GLUL inhibition (*p* < 0.01) compared to Dicer 2A (Figure 9C,D). Furthermore, gemcitabine sensitivity was also significantly increased (*p* < 0.0001) (Figure 9E,F). Therefore, the above results further confirm that the phosphorylation of Dicer at S1728E/S1852E leads to the development of gemcitabine resistance and affects cancer cell proliferation by altering glutamine metabolism balance.

### 2.6. Phosphorylated Dicer Promotes Competitive Inhibition Between Pyrimidine Synthesis-Related Enzymes and the Metabolites Produced by Gemcitabine, Leading to the Development of Gemcitabine Resistance

Previous results showed that Dicer 2E cells had significantly higher concentrations of glutamine and tended to absorb more glutamine from the medium into the cells. Furthermore, Dicer 2E cells also had higher GLUL expression than other cells, indicating that Dicer 2E does not tend to convert intracellular glutamine to glutamate but has another purpose. Considering the perspective of cancer metabolism abnormalities, cancer cells need to rely on glutamine to increase nucleotide synthesis, including purines and pyrimidines, to support and promote cancer cell proliferation. However, the pharmacological mechanism of gemcitabine is to produce nucleoside analogs, preventing DNA synthesis. Since nucleotide synthesis requires glutamine, Dicer 2E may tend to use glutamine for pyrimidine synthesis, further reducing the efficacy of gemcitabine and simultaneously increasing the proliferation of cancer cells.

**Figure 10:**
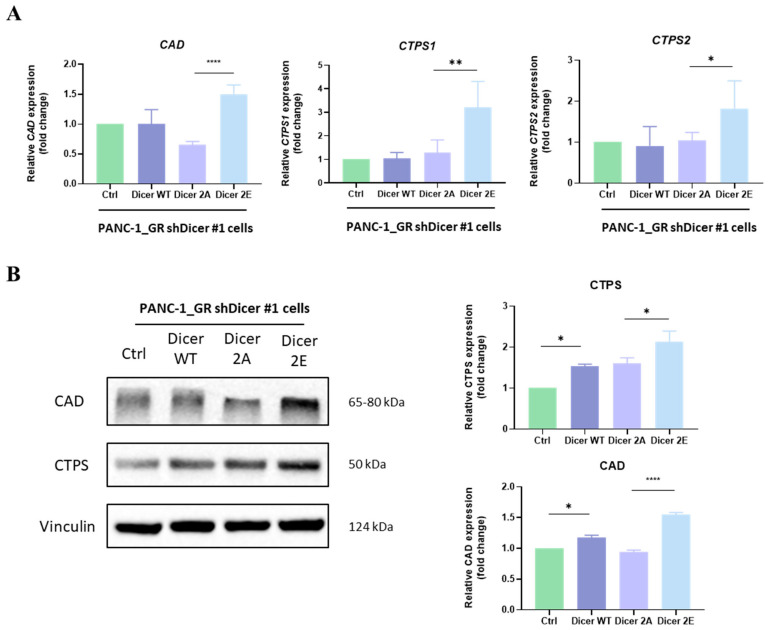
Simulating phosphorylation of Dicer at S1728E/S1852E increases the expression of enzymes CAD and CTPS1 and 2 required for pyrimidine synthesis, leading to competitive inhibition by gemcitabine’s end metabolites and resulting in gemcitabine resistance. (**A**) The mRNA expression levels of CAD and CTPS1 and 2 in Ctrl, Dicer WT, Dicer 2A, and Dicer 2E cells were tested using qRT-PCR. The qRT-PCR data were normalized to the GAPGH level in each sample, and a bar plot presents fold changes in the expression of PANC-1_GR_shCtrl#1 cells. (**B**) The protein expression levels of CAD and CTPS1 and 2 in Ctrl, Dicer WT, Dicer 2A, and Dicer 2E cells were tested using Western Blot, with Vinculin as the control. Data are presented as mean ± SEM and analyzed using One-way ANOVA. Significant differences are indicated when *p* < 0.05 (represented as * *p* < 0.05, ** *p* < 0.01, and **** *p* < 0.0001).

We examined the mRNA expression levels of enzymes related to pyrimidine synthesis in Dicer 2E cells, including CAD (carbamoyl-phosphate synthetase 2, aspartate transcarbamylase, and dihydroorotase) and CTPS1 and 2 (cytidine triphosphate synthetase). Consistent with previous results, Dicer 2E indeed showed significantly higher mRNA expression levels of these enzymes (*p* < 0.05) (Figure 10A). Further, through Western Blot analysis, the protein expression levels of these pyrimidine synthesis-related enzymes in Dicer 2E were also significantly increased (*p* < 0.01) (Figure 10B). Therefore, it is reasonable to infer that the phosphorylation at the Dicer S1728 and S1852 sites can promote pyrimidine synthesis by utilizing the accumulated intracellular glutamine, thereby reducing the efficacy of gemcitabine and increasing cancer cell proliferation.

## 3. Discussion

In PANC-1 GR/shDicer cells, Dicer wild type (WT), Dicer 2A (S1728A, S1852A), and Dicer 2E (S1728E, S1852E) were overexpressed. The central concept is to mutate the serine (S) residues at positions 1728 and 1852 of Dicer to alanine (A) to simulate a phosphomutant state and to mutate them to glutamine (E) to simulate a phosphomimetic state. It was found that Dicer 2E cells promoted cancer cell proliferation and led to drug resistance in pancreatic cancer-resistant cells. Previous clinical studies have also found that the phosphorylation level of Dicers S1728 and S1852 positively correlates with the invasion degree of primary endometrial cancer [9]. Additionally, mouse experiments have shown that phosphorylated Dicer S1728 and S1852 cells can cooperate with KRASG12D to increase the likelihood of tumor formation, such as lung adenocarcinoma and lymphoma, while also significantly reducing mouse survival rates [10]. In this experiment, the cell line PANC-1 is a KRASG12D PDAC cell line. Previous literature indicates that KRAS mutations and activation are critical genetic drivers of PDAC occurrence and progression and are crucial for maintaining PDAC tumor growth [16,17]. The RAF–MEK–ERK pathway is its downstream regulatory factor [18]. Combined with previous research in our laboratory, it was found that the ERK-induced SP1 transcription factor binds to the Dicer promoter region, increasing Dicer expression, which helps the survival of PDAC cells resistant to gemcitabine [8]. These results suggest that the upstream re-mechanism of phosphorylated Dicer may be synergistic with KRASmut in PDAC, driving the development of drug resistance.

Regarding whether phosphorylation of Dicer at different sites has different effects, compared to previous research in our laboratory, it was found that phosphorylation of Dicer at S1016 in pancreatic cancer cells resistant to gemcitabine also regulates specific miRNAs, affecting glutamine metabolism and leading to gemcitabine resistance. However, its effect decreases GLUL expression, increasing the GLS/GLUL ratio [11]. This result indicates that pancreatic cancer cells tend to convert glutamine to glutamate. However, further experiments confirm whether the downstream mechanism involves the enzyme GLUD1 or various mitochondrial transaminases GPT2 and GOT2 to convert glutamate to α-KG and enter the TCA cycle to assist the Warburg effect [19]. What is certain so far is that the phosphorylation of Dicer at different sites primarily affects glutamine metabolism, influencing the drug sensitivity of cancer cells to gemcitabine. It may regulate different enzymes in the metabolic pathway, driving glutamine to perform different functions within the cell. More research is needed to confirm the differences in these pathways. Additionally, whether other different sites also influence the development of drug resistance through different pathways is worth exploring. In this era of precision medicine, not only can we judge the degree of drug resistance through the level of phosphorylated Dicer in tumor tissues of different patients, but we can also provide appropriate adjuvant therapy based on the phosphorylation of Dicer at different sites.

MicroRNA (miRNA) is classified as non-coding mRNA, with about 22 long nucleotides. Due to its impact on gene expression, abundance in body tissues and fluids, and potential use as disease biomarkers, miRNA is a significant area of basic and translational biomedical research. This study showed that phosphorylated Dicer 2E cells had significantly higher expression levels of miR-140-5p and miR-29a-5p than Dicer 2A and Dicer WT cells (Figure 8A,B). Both miRNAs are predicted to bind to the 3′-UTR of GLUL, further affecting its expression. This result is consistent with the GLUL mRNA expression levels in Dicer 2E cells (Figure 6B). Previous studies have also confirmed that miR-140-5p and miR-29a-5p can bind to the 3′-UTR of GLUL, affecting the invasion and proliferation of glioma cells [20,21]. Additionally, concerning how phosphorylated Dicer regulates specific miRNAs, previous literature indicates that under hypoxic conditions expected in the tumor microenvironment, the epidermal growth factor receptor (EGFR) can phosphorylate argonaute (AGO) at Y393, changing its structure. The phosphorylated side chain structure protrudes towards the cavity between the N-structure domain (EGFR’s interaction surface) and the L2 linker region (the linker domain between PAZ and MID), affecting the interaction between Dicer and AGO and altering the maturation of specific miRNAs. Phosphorylated Dicer may also influence its structure, further affecting the maturation of particular miRNAs [22]. However, researchers have different speculations and concerns about miRNAs. Due to their low specificity, many miRNAs can act as oncogenes (oncomiRs) or tumor suppressor genes (oncosuppressor miRs), and dysregulated miRNA expression is closely related to the occurrence, progression, and metastasis of cancer [23,24]. Therefore, regarding the choice of miR-140-5p as a research target, previous literature indicates that its expression is significantly lower in most cancers. For example, in esophageal cancer, low expression of miR-140-5p can regulate ZEB2 expression to block Wnt/β-catenin signaling, further affecting cancer cell proliferation, invasion, and metastasis [25]. In gastric cancer, low expression of miR-140-5p can regulate the EMT regulator SOX4 to inhibit cancer cell proliferation and metastasis [26]. On the other hand, miR-140-5p fundamentally influences cancer cell sensitivity to chemotherapy or radiotherapy. For example, low expression of miR-29a-5p in gastric cancer can regulate NDRG3, further increasing cancer cell resistance to the chemotherapeutic drug 5-fluorouracil [27]. Additionally, other literature has observed that overexpression of miR-29a-5p in PDAC cells can inhibit cancer cell proliferation and invasion [28]. For example, enhanced expression of miR-29a can inhibit mucin 1 (MUC1), reducing the expression levels of cell cycle-dependent kinases CDK2, CDK4, and CDK6, leading to reduced proliferation of gastric cancer cells [29]. The results of this study, combined with the above literature, support that miR-140 and miR-29a can act as inhibitors regulating cancer cell proliferation.

Additionally, we used the GLUL inhibitor methionine sulfoximine (MSO), an organic sulfur analog of glutamate and an irreversible competitive inhibitor of GLUL, to effectively inhibit GLUL activity. The results showed that after administering MSO, PDAC cells’ sensitivity to gemcitabine increased, especially in Dicer 2E cells with significantly higher GLUL expression levels (Figure 6B). Conversely, Dicer 2A and Dicer WT cells responded less to MSO, consistent with their lower GLUL expression levels (Figure 6B). Previous literature has discussed the potential of GLUL inhibitors as cancer treatment drugs. Due to the highly fibrotic characteristic of pancreatic ductal adenocarcinoma [30], the supply of glutamine in cancer cells is limited, potentially leading to glutamine deficiency [31]. Therefore, increased GLUL expression in pancreatic cancer patients and PDAC mouse models supplies glutamine to cancer cells to prevent glutamine deficiency [30]. Additionally, GLUL plays an important role not only in cancer cells but also in cells in the tumor microenvironment (TME), including cancer-associated fibroblasts (CAFs) and macrophages, where GLUL expression is also higher. When glutamine is insufficient in cancer cells, GLUL provides glutamine to support cancer cell proliferation. Therefore, GLUL is an essential option for cancer treatment targeting the specificity of the pancreatic cancer tumor microenvironment. Conversely, other literature indicates that silencing GLUL expression inhibits nucleotide synthesis, suppresses tumor growth in LSL-KrasG12D/+; Pdx1-Cre (KPC) PDAC mice, and increases survival rates [32]. These findings indicate that GLUL and nucleotide synthesis are crucial for PDAC tumor development and growth. Additionally, this study found that in the absence of glutamine, phosphorylated Dicer 2E cells’ sensitivity to gemcitabine significantly increased, and cancer cell proliferation significantly decreased, indicating that glutamine is indeed an essential material for drug resistance and cell proliferation (Figure 9A,B). Previous literature also suggests that administering a glutamine analog (6-diazo-5-oxo-L-norleucine, DON) to the pancreatic cancer-resistant cell line MiaPaca-2 GR significantly increases sensitivity to gemcitabine [14]. These findings further confirm that PDAC cells’ resistance to gemcitabine is closely related to glutamine metabolism.

This study found that Dicer 2E cells, compared to Dicer 2A cells, significantly increased the expression of glutamine transporter proteins (SLC1A5, SLC1A5_var, and SLC38A1) (Figure 6C–E). This result is consistent with the substantially higher glutamine concentration in Dicer 2E cells (Figure 7C). Previous experiments also found significantly higher expression levels of alanine–serine–cysteine transporter 2 (ASCT2, also known as SLC1A5), SLC1A5_var, and SLC38A1 in PDAC cells and tissues. Silencing these genes affects glutamine metabolism and further influences cancer cell proliferation, even leading to apoptosis [19,33,34]. Additionally, other literature indicates that miR-122-5p can bind to SLC1A5 in PDAC, regulating its expression to alter glutamine metabolism [35]. This also suggests that the gene expression observed in phosphorylated Dicer S1728/S1852 might be regulated by specific miRNAs, which requires further experiments to confirm.

CAD (carbamoyl-phosphate synthetase 2, aspartate transcarbamylase, and dihydroorotase), CTPS1, and CTPS2 (cytidine triphosphate synthetase) are enzymes required for pyrimidine synthesis, a process that involves glutamine. This experiment observed that Dicer 2E cells had significantly higher intracellular glutamine concentrations compared to Dicer 2A cells (Figure 7C), consistent with the higher glutamine consumption in Dicer 2E cells (Figure 7A). However, they convert glutamate to glutamine (Figure 6B). Further, it was found that Dicer 2E tends to use accumulated intracellular glutamine for pyrimidine synthesis, leading to gemcitabine resistance (Figure 10A,B). Previous studies have also found that in gemcitabine-resistant pancreatic cancer cell lines, pyrimidine synthesis increases, leading to gemcitabine resistance through the HIF-1α-induced aerobic glycolysis pathway [36]. Therefore, whether the phosphorylation of Dicer at S1728 and S1852 involves other mechanisms to increase pyrimidine synthesis and lead to drug resistance remains to be clarified.

## 4. Materials and Methods

### 4.1. Cell Culture

The cell line used in this study was PANC-1. For different experiments, 10 cm dishes, 6-well plates, and 96-well plates were used, and the cells were cultured in an incubator at 37 °C with 5% CO_2_. To establish a gemcitabine-resistant PANC-1 pancreatic cancer cell line, PANC-1 cells were treated with 0.5 µM, 1 µM, and 2 µM concentrations of gemcitabine. The surviving cells were selected and passaged, resulting in a cell line capable of surviving at a gemcitabine concentration of 2 µM. Once stabilized, these cells were termed gemcitabine-resistant cells (PANC-1_GR). The MTT assay was used to verify the drug resistance of the resistant cell line and to assess the differences in survival rates at various gemcitabine concentrations.

Both the PANC-1 and PANC-1 GR cells were maintained in Dulbecco’s Modified Eagle’s Medium (DMEM, 23-10-013-CM, Corning^®^, Corning, NY, USA) containing high glucose (4500 mg/L), l-Glutamine (4 mM), and sodium pyruvate (1 mM) with 10% fetal bovine serum and 1% penicillin–streptomycin. The cells were incubated at 37 °C with humidified 5% CO_2_, and the medium was replaced every 3 days until 70% confluency was reached.

### 4.2. Cell Viability Assay

The cells were seeded in a 96-well plate (approximately 3000 cells per well). After 24 h, the test drugs, such as gemcitabine and MSO, were added. At 24, 48, and 72 h, 50 µL/well of 3-(4,5-dimethylthiazol-2-yl)-2,5-diphenyltetrazolium bromide (MTT) solution (1 μg/mL) was added. After 4 h, the culture medium was removed, and 100 µL/well of DMSO was added to dissolve the purple crystals. The plate was covered with aluminum foil and shaken for 15 min. The absorbance at 570/630 nm was then measured using an EPOCH2 microplate spectrophotometer, and cell viability was expressed as a percentage of viable cells relative to the control group (set at 100%) and analyzed using GraphPad software.

The cells were seeded into a 6-well plate (approximately 500 cells per well) and placed in a 37 °C, 5% CO_2_ incubator. Different drug concentrations were added the next day according to the experimental design. After one week, the culture medium was removed and the cells attached to the bottom were fixed with 10% formalin and subjected to shaking using a shaker for 30 min. Then, the cells were stained with 0.5% crystal violet and subjected to shaking using a shaker for another 30 min. After washing thoroughly, images were captured using a digital camera. Finally, the images were analyzed using Image J software (Version 1.54k).

### 4.3. RNA Isolation and Quantitative RT-PCR

Add 200 µL of sterile DEPC-treated H_2_O to the cells intended for RNA extraction, mix thoroughly, then add 500 µL of NucleoZol (REF 740404.200, Macherey-Nagel, Düren, Germany). Next, add an equal volume of isopropanol and centrifuge, and then remove the supernatant. Wash the RNA pellet gently with 500 µL of 75% ethanol, and then discard the supernatant, leaving the RNA pellet. Finally, the pellet should be dissolved by adding an appropriate amount of sterile DEPC-treated H_2_O according to the volume of the RNA pellet. The extracted RNA is then added to RNA reverse transcription reagents (Invitrogen, Waltham, MA, USA) to convert it into cDNA. Quantitative RT-PCR (qRT-PCR) measurements use the Lightcycler 480 system (Roche, Basel, Switzerland). Primer sequences used in the lab are designed based on the ROCHE Universal Probe Library Assay Design Center and NCBI database.

### 4.4. Western Blotting

Add the cell lysis buffer (980 µL RIPA buffer, 10 µL PIC (10X), 10 µL 20 mM Na_3_VO_4_) to the cells and use an ultrasonic cell disruptor to sonicate the samples. Using the Bradford protein-binding assay, use a centrifuge to collect the supernatant-containing proteins and measure the protein concentration. Then, perform sodium dodecyl sulfate-polyacrylamide gel electrophoresis and transfer the proteins to polyvinylidene difluoride membranes. Next, soak the membrane in 5% blocking buffer (2.5 g skimmed milk powder and 50 mL TBST) for one hour, and incubate overnight at 4 °C with the indicated primary antibodies: GAPDH (1:7500; ABclonal, Cambridge, MA, USA) and Dicer (1:1000; ab14601, Abcam, London, UK). Wash the membrane with TBST and incubate for 1 h at room temperature with appropriate secondary antibodies conjugated to horseradish peroxidase. Subsequently, visualize the bands on the membrane using enhanced chemiluminescence (ECL) and capture the images using the UVP ChemiDoc-IT 515 Imaging System and Vision Work software (81-0225-01 Rev K).

### 4.5. Lentiviral Knockdown and Phosphomimetic Dicer Constructs

The pLKO.1-puro-based lentiviral vectors TRCN0000290426 (shDicer#1) and the control plasmids TRC025.shLKO (shCtrl#1) were purchased from the National RNAi Core Facility at Academia Sinica, Taipei, Taiwan. HEK293T cells were cotransfected with the lentivirus expression plasmid, packaging plasmid (pCMV-dR8.91), and envelope plasmid (VSV-G expressing plasmid, pMD2.G) with polyethyleneimine (Merck, Rahway, NJ, USA) for 48 h. We generated a recombinant lentivirus from the culture medium. The cells were infected with lentiviruses combined with 8 μg/mL polybrene, and stable cells were selected using 2 μg/mL puromycin.

To overexpress Dicer and phosphomimetic Dicer, the Dicer WT plasmid (pCAGGS-Flag-hsDicer plasmid #41584) was purchased from Addgen, and then Dicer was subcloned into the pcDNA6/myc-His vector. The pcDNA6-Dicer S1728A/1852A and S1728E/1852E mutations were achieved using the Q5 site-directed mutagenesis kit (NEB E0554S). The cells were transfected with a Dicer WT, S1728A/S1852A or S1728E/S1852E plasmid, or a control vector (pcDNA6) for 48 h by using the NTR II (non-liposome transfection reagent), JT97-N002, T-Pro Biotechnology, following the manufacturer’s instructions, and stable cell lines were selected using 4 μg/mL blasticidin (ant-bl-05; InvivoGen, San Diego, CA, USA).

### 4.6. Glutamine Consumption and Glutamate Secretion Assay

Seed cells into a 96-well plate (approximately 3000 cells per well) and place in a 37 °C, 5% CO_2_ incubator. After 72 h, take 2 µL of the culture medium and add 98 µL of DPBS to dilute it, resulting in the sample. Add 25 µL of the sample to 25 µL of glutaminase enzyme solution or buffer (glutamine/glutamate-Glo assay kit, Promega Corporation, Madison, WI, USA) and shake using a shaker at room temperature for 30 to 60 s. Then, incubate in the dark at room temperature for 30 to 40 min and measure using a Microplate Reader, Thermo Varioskan Flash, Waltham, MA, USA.

### 4.7. Statistical Analysis

GraphPad Prism version 8 was used as the statistical software for creating charts. All data are presented as mean ± standard error of the mean (mean ± SEM). Differences between the two experimental groups were compared using Student’s *t*-test. One-way analysis of variance (One-way ANOVA) was used to compare the mean differences among three or more groups with a single variable, and Two-way analysis of variance (Two-way ANOVA) was used to compare the mean differences among three or more groups with two variables. The results are considered statistically significant when *p* < 0.05.

## 5. Conclusions

This study mainly discovered that the occurrence of drug resistance and the promotion of cancer cell proliferation in pancreatic ductal adenocarcinoma are closely related to the phosphorylation of the Dicer S1728/S1852 sites. It was found that phosphorylated Dicer S1728E/S1852E can lead to abnormal glutamine metabolism by regulating the expression levels of miR-140-5p and miR-29a-5p, thereby increasing intracellular glutamine concentration and promoting pyrimidine synthesis. This results in competitive inhibition with the final metabolites of gemcitabine, leading to gemcitabine resistance. In the future, phosphorylated Dicer S1728/S1852 could indicate drug resistance in pancreatic ductal adenocarcinoma patients, allowing for appropriate adjuvant therapy based on the degree of its phosphorylation.

## Data Availability

The data used in this study are available from the corresponding author upon reasonable request.
